# Odor response adaptation in *Drosophila*—a continuous individualization process

**DOI:** 10.1007/s00441-020-03384-6

**Published:** 2021-01-25

**Authors:** Shadi Jafari, Mattias Alenius

**Affiliations:** 1 Department of Biology, New York University, New York, NY USA; 2 Department of Molecular Biology, Umeå University, 901 87 Umeå, SE Sweden

**Keywords:** Drosophila, Olfactory perception, Odorant

## Abstract

Olfactory perception is very individualized in humans and also in *Drosophila*. The process that individualize olfaction is adaptation that across multiple time scales and mechanisms shape perception and olfactory-guided behaviors. Olfactory adaptation occurs both in the central nervous system and in the periphery. Central adaptation occurs at the level of the circuits that process olfactory inputs from the periphery where it can integrate inputs from other senses, metabolic states, and stress. We will here focus on the periphery and how the fast, slow, and persistent (lifelong) adaptation mechanisms in the olfactory sensory neurons individualize the *Drosophila* olfactory system.

## OSN adaptation sites

Odorant or pheromone molecules bind chemoreceptors localized on specialized signaling organelles of olfactory sensory neurons (OSNs) called cilia (Benton et al. 2006). Insects have three different types of chemoreceptors: odorant receptors (ORs, Clyne et al. 1999; Vosshall et al. 1999), gustatory receptors (GRs, Scott et al. 2001), and ionotropic receptors (IRs, Benton et al. 2009). Most OSNs express one odor-specific OR (Couto et al. 2005) along with the olfactory co-receptor Orco (Larsson et al. 2004), which is highly conserved across insect species (Jones et al. 2005). Each of the 20–100 OSNs that express the same receptor form synapses in the antennal lobe of the brain with an average of three excitatory projection neurons (PNs) and local interneurons (LNs) (Ng et al. 2002). The synapses of each OSN class together form one of 52 separate glomeruli with stereotypic location, shape, and size in the *Drosophila* antennal lobe brain region (Couto et al. 2005; Fishilevich and Vosshall 2005). Each class of OSNs localize to restricted antenna regions. Like this, the odorant plumes reach all OSNs in a class almost simultaneously, leading to synchrony in OSN responses. The convergence coupled with synchrony in olfactory responses forms a coincidence filter that increases the signal-to- noise ratio (Ng et al. 2002).

Adaptive restrictions on olfactory transduction and OR response thresholds broaden the informative odorant concentration range and reduce information flow through olfactory circuits. Only ORs, but not GRs or IRs, seem subject to adaptation (Getahun et al. 2012). Beside odor transduction, synapse activity adaptation is the major adaptation site in OSNs and the first with direct retrograde input from the brain.

## Fast adaptation

The fast adaptation process is cell autonomous and last minutes. The fast mechanisms divide into short and long term. Short-term adaptation acts within seconds and fine tunes transduction and synapse activity (Cafaro 2016; Kadakia and Emonet 2019; Nagel and Wilson 2011). Long-term adaptation responds to the background odorant level and suppresses transduction on a time scale of multiple seconds (Table 1, Martelli and Fiala 2019).

## Short-term fast adaptation

**Fig. 1:**
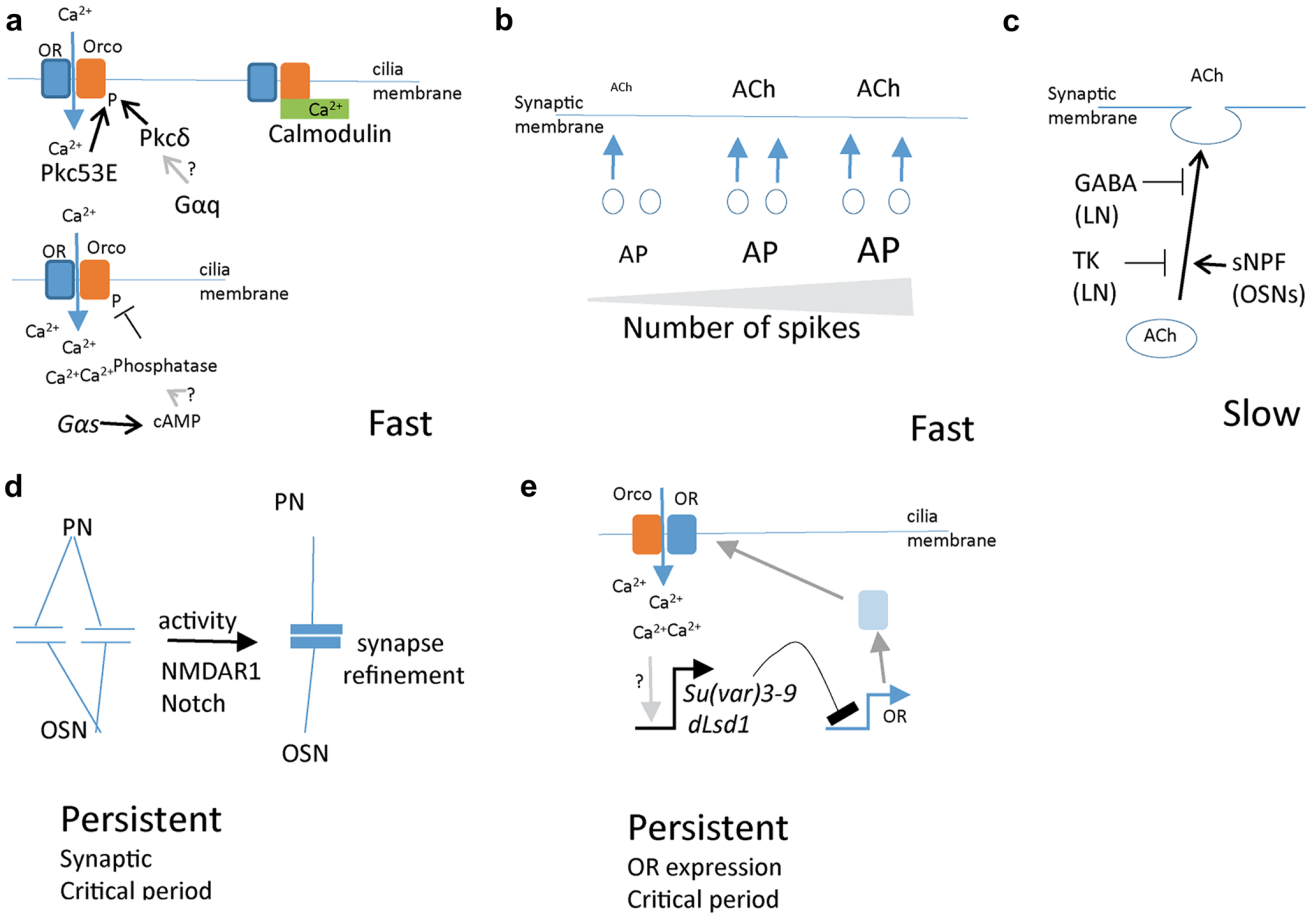
Direct feedback mechanism

Short-term fast adaptation influences OR channel opening times and proceeds through the olfactory co-receptor Orco (Getahun et al. 2012). A brief odor stimulus activates PKC, which phosphorylates Orco and increases odor responses (Fig. 1a, Getahun et al. 2016; Guo et al. 2017). This phosphorylation of Orco by PKC is required for odor source localization in flying *Drosophila* (Getahun et al. 2016; Guo et al. 2017), supporting its role as a mechanism of short-term acute adaptation. In addition to its phosphorylation sites, Orco contains a conserved calmodulin interaction motif (Getahun et al. 2016) that supports ciliary transport of Orco in a direct feedback mechanism (Fig. 1a, Bahk and Jones 2016; Mukunda et al. 2014; Mukunda et al. 2016). It remains to be established whether these PKC-mediated and calmodulin-mediated mechanisms are related.

Of the five PKCs in *Drosophila*, *Pkc53E* and *Pkcδ* increase OR sensitivity (Getahun et al. 2016). Calcium regulates Pkc53E, but not Pkcδ, which is downstream of GPCR signaling (Lipp and Reither 2011). Brief odor responses induce GPCR signaling, as *Gqα* subunit mutant flies have reduced odor response kinetics (Kain et al. 2008). *Drosophila* ORs are seven transmembrane but with a reversed topology and it is debated if they are GPCRs (Benton et al. 2006). One alternative GPCR candidate is the Hedgehog pathway. Hedgehog signals through the GPCR smoothened, which regulates PKC (Teperino et al. 2014). Smoothened is localized to OSN cilia where it regulates OR transport to the ciliary membrane (Kuzhandaivel et al. 2014; Sanchez et al. 2016). OSNs release Hedgehog, which increases odor responses (Sanchez et al. 2016). Whether the release of Hedgehog is coupled to OSN activity remains to be investigated.

## Long-term fast adaptation

Strong and prolonged odor responses dephosphorylate Orco and suppress subsequent odor responses (Guo et al. 2017). The kinetics of this phenomenon indicate a tight link to activity and suggest a calcium-induced phosphatase (Fig. 1a), but the responsible phosphatase is not yet identified. Interestingly, mutants of another G-protein, *Gαs*, show delayed odorant response desensitization (Deng et al. 2011), and odor response kinetics also affect cAMP levels (Miazzi et al. 2016; Smart et al. 2008). This suggests that different GPCR pathways may act in opposition to balance fast olfactory adaptation.

Activity at OSN/PN synapses also shows fast adaptation. PNs respond strongest to small, sharp changes in signaling from OSNs rather than to the overall size of the response (Nagel and Wilson 2011). This non-linearity arises from the limited transmitter vesicle pool available in OSN terminals; a short spike train releases all the available transmitter and any subsequent increase in spike production releases less transmitter (Fig. 1b). This increased sensitivity to change allows PNs to respond with faster kinetics than OSNs.

## Slow adaptation

Slow adaptation contrasts to fast adaptation being non-cell autonomous and can shift responses between OSN classes (Table 1). The slow adaptation primarily modulates presynaptic transmission, but extended odor exposures also lead to post-synaptic PN adaptation (Cafaro 2016; Nagel et al. 2015). GABA signaling underlies most slow adaptation (Fig. 1c, Olsen and Wilson 2008; Wilson and Laurent 2005). GABAergic LNs form synapses with OSNs (Olsen and Wilson 2008; Root et al. 2008; Wilson and Laurent 2005), and the various OSN classes differ in GABA sensitivity (Grabe et al. 2020; Hong and Wilson 2015). The size of the GABA response relates to the size of the odor response, with larger responses acting to dampen extreme OSN/PN activity (Grabe et al. 2020; Olsen and Wilson 2008). This suppression results in a gain control mechanism that increases the dynamic range of information transmission (Olsen and Wilson 2008; Root et al. 2008; Wilson and Laurent 2005).

GABAergic LNs also produce the neuropeptide tachykinin (TK) (Ignell et al. 2009). High levels of OSN activity increase TK release (Winther and Ignell 2010), causing the TK receptor signal to mediate presynaptic hyperpolarization (Fig. 1c), which then contributes to presynaptic gain control (Ignell et al. 2009). OSNs also release a neuropeptide, sNPF, that, in contrast to GABA and TK signaling, increases presynaptic calcium responses (Fig. 1c, Root et al. 2011). The OSN *sNPF* and the *GABABR2* receptor expression overlap partially (Carlsson et al. 2010). Skewing the balance between GABA and sNPF signaling can thus alter OSN inputs to the antennal lobe. During starvation, OSNs increase the expression of the sNPF and TK receptors, which enhance the contrast between negative and positive odor responses and increase attraction to food odors (Ko et al. 2015; Mohamed et al. 2019). If activity regulates sNPF and balances the OSN activity level remains to be addressed.

## Critical periods, a lifelong persistent adaptation

Adult flies emerge with a naïve sensory system whose receptor expression and synaptic activity levels are not necessarily in sync with the environment. Coarse tuning of many sensory systems takes place during a restricted “critical period” (Hensch 2004). Critical periods for adaptation are distinguished from ongoing synaptic plasticity by the following criteria: they (i) have a distinct time and duration, (ii) are sensitive to experience and neuronal activity, (iii) lead to permanent changes, (iv) induce competition within the system that generates refinement, and (v) have a defined mechanism (Hensch 2004). *Drosophila* OSNs have two parallel and likely unrelated critical periods, one that refines OSN/PN synapses (Fig. 1d) and another that adjusts OR levels (Fig. 1e, Table 1, Devaud et al. 2001; Golovin et al. 2019; Jafari and Alenius 2020).

## Critical period for OSN synapse formation

OSNs make permanent, activity-dependent changes to their synaptic connections with PNs and LNs during the first 3 days of adult life (Devaud et al. 2001). Studies activating a specific OSN class show distinct changes from studies where the activity comes from another OSN class (Fig. 1d, Devaud et al. 2001; Golovin et al. 2019). This suggests an interdependence between OSN classes, but direct competition between glomeruli has not yet been addressed. Nevertheless, OSN/PN synaptic adjustments are cell autonomous and require both NMDAR1/glutamate and Notch signaling (Golovin et al. 2019; Kidd et al. 2015). The OSN/PN critical period is followed by extensive adaptation and another critical period for the refinement of LN/PN synapses (Chodankar et al. 2020; Das et al. 2011; Sachse et al. 2007). These synaptic critical periods thus balance inputs and outputs.

## Critical period for OR expression

OR expression in *Drosophila* is generally considered a static (Imai et al. 2010), predetermined process, but there is considerable plasticity in the levels of OR, GR, and IR expression in the antenna for the first 2 days of an adult fly’s life (Jafari and Alenius 2020). This chemoreceptor expression plasticity also fulfills the criteria for a critical period. In these first 2 days, not only does the odor environment modulate OR expression (Iyengar et al. 2010; Koerte et al. 2018; von der Weid et al. 2015), but OR overexpression can also suppress endogenous OR expression (Fig. 1e, Jafari and Alenius 2020). This indicates a direct competition between ORs in the regulation of their expression. Interestingly, activity regulates odorant-responsive and pheromone-responsive OSNs differently (Jafari and Alenius 2020). OR expression is also stress-sensitive, with the stress-induced changes becoming permanent if the stress lasts beyond the critical period. Thus, both stress and environmental odors set the baseline for OR expression throughout a fly’s life.

The mechanisms underlying the OR gene regulatory critical period build on heterochromatin regulation in ways that are similar to the OR choice mechanism in mouse olfactory neurons (Jafari and Alenius 2020; Monahan and Lomvardas 2015). In non-OSNs in *Drosophila*, OR genes are embedded in heterochromatin marked with H3K9me3 produced by Su(var)3-9, which prevents OR expression (Gonzalez et al. 2019; Jafari and Alenius 2015; Sim et al. 2012). Consistent with this, the H3K9me3 erasing enzyme Kdm4b, which open heterochromatin, initiates OR expression. A second enzyme, dLsd1, removes the remaining H3K9 methylation and further establishes OR expression (Jafari and Alenius 2020). OR activity induces and balances these enzymes to modulate OR expression (Fig. 1e, Jafari and Alenius 2020) until a balance between activity and OR expression level is achieved. Thus, this feedback system functions as a rheostat that ensures olfactory responses to be within physiological limits, avoiding hyperactivation of the olfactory system. Stress suppresses activity-induced heterochromatin formation and promotes OR expression. The fact that a similar feedback mechanism is found in vertebrates (Abdus-Saboor et al. 2016; von der Weid et al. 2015) and that OR activity suppress OR expression also in mosquitoes (Maguire et al. 2020) suggests that environmental odors and stress can set OR expression baselines across phyla. A clearer picture of the mechanisms that maintain OR expression in the adult is required to understand how the acute and extended adaptation pathways set, maintain, and change the OR expression baseline.

## Concluding remarks and future perspectives

Olfactory adaptation determines the beginning of each new olfactory percept and influences future percepts as well. Each adaptation step reflects the fly’s earlier olfactory history. From the coarse early refinement to the continuous real-time adjustments, these adaptive mechanisms individualize a fly’s olfactory responses. To limit these differences in adaptation and minimize variability in our studies, we use standardized fly housing and handling conditions. Thus, a feasible next step is to introduce specific variabilities and define how previous exposure to odor or stress alter the acute effects.

Several technical aspects must be considered when attempting to further study the mechanisms of olfactory adaptation. Thus far, most adaptation studies focused on a few OSN classes. Now, with the development of sensitive calcium and chloride imaging techniques, presynaptic adaptation can be analyzed across all classes at a millisecond time scale. Large-scale transduction studies have been limited by the array of mixed OSN classes on the antennal surface combined with the low spatial resolution of imaging techniques. Nevertheless, tour de force studies have recently pushed back at these limitations (Grabe et al. 2020; Seki et al. 2017). Ultimately, future studies must provide the necessary spatial resolution to observe the filtering that takes place between OSN classes at all stages of adaptation.

The *Drosophila* model itself is also a major technical hurdle that must be overcome. The many genetic tools developed in *Drosophila* lack the temporal resolution to match the kinetics of olfactory adaptation. Acute adaptation takes place on the scale of milliseconds to minutes, while existing temporally controlled genetic techniques function over the course of days. Alternative tools that can follow the process live may solve some of these many temporal issues. The phospho-Orco antibody (Guo et al. 2017), for example, shows that the next generation of tools need not be driven by high technology, just well-suited to the specific mechanism of interest. An alternative is machine learning algorithms and mathematical models of electrophysiological measurements (Kadakia and Emonet 2019; Nagel et al. 2015), which may help bridge the static results of genetic studies and the dynamics of adaptation.

Here, we have discussed how OSNs, rather than being mere input channels, represent a filter and that the filter is prone to changes. Therefore, to understand the central olfactory processing, the peripheral filtering of olfactory inputs at the level of the OSNs must be taken into account and understood. Thus, the time has come to tweak our assays to get a richer picture of the olfactory life of *Drosophila* and other species.

**Table 1:** Background odorant level

	Initiation	Duration	Tuning	Cell autonomous	Adapted step	Mechanism
Critical period (OR expression)	First days in life	Weeks	Coarse baseline formation	Yes	Transduction	OR expression modulation
Critical period (synapse refinement)	First days in life	Weeks	Coarse baseline formation	No	Synapse	Synapse stability
Slow	Minutes to hours after response	Hours to days	Modulation	No	Synaptic activity	Negative: GABA, TK positive: sNPF
Fast (long term)	Immediate	Seconds to minutes	Fine	Yes	Transduction	OR/Orco cilia transport
Fast (short term)	Immediate	Seconds	Fine	Yes	Transduction	Orco phosphorylation
